# The Abuse of Mercury in the Treatment of Eye Diseases

**Published:** 1892-04-09

**Authors:** 


### OPHTHALMOLOGY.

### THE ABUSE OF MERCURY IN THE TREATMENT OF EYE DISEASES.

In a recent communication on the abuse of mercury in the treatment of diseases of the eye, Professor Landolt*, of Paris, remarks that we too willingly share the belief that a merciful Providence has provided us with an antidote found in the vegetable or mineral kingdom for every malady, and that the administration of these drugs or remedies has become a sort "of auto-suggestion, a reflex action which is produced by the sight only of the pathological symptoms." Believing in a remedy we are too apt to neglect searching diligently for one which may be more efficacious.

Such is the case with mercury, a drug which, though of the greatest use in suitable cases, is far too often administered without due consideration of the pathology of the disease under observation. He points out how often it is prescribed as it were, by rule of thumb, in cases in which all hope of cure is dissipated by the fact that there is pathological destruction of tissue, as seen by aid of the microscope, and specially mentions the optic atrophy symptomatic of tabes

* British Medical Journal, for March 26th, 1892.

dorsalis, in which he asserts that even if the disease be due to syphilis it represents such an advanced stage of it that mercury can have but little chance of modifying it. On this question we know that high authorities differ in opinion, some holding that mercury, even in the later stages of syphilis, is a most useful adjunct to treatment, though as far as Professor Landolt's objection to it as a cure for the optic atrophy goes, they would doubtless agree with him. The pathology of a large number of diseases of the eye is not clearly known to us, but in those in which there is great destruction of tissue, he points out that mercury cannot restore their functions.

He remarks that it is needless to say that in ocular affections engendered by syphilis, the appropriate treatment for such a diathesis—and amongst others, mercury—is absolutely indicated. But it is the reckless administration of it, in cases not certainly arising from syphilis, that he objects to it. In some text books, for instance, there is an axiom, "*In cases of iritis always prescribe mercury at the first visit.*" He, however, holds that mercury is quite unnecessary in iritis, and quotes a case of exaggerated iritis, in which a complete cure was obtained without the administration of mercury.

There is no doubt that mercury is often harmfully administered with the best intentions; a body already emaciating from under-feeding, struma, and mental anxiety, as well as pain from the eye complaint, is not likely to be improved by the administration of mercury, which is prone to produce anæmia, though when the anæmia is due to syphilis, which is a powerful disintegrator of red corpuscles, mercury acts as an arresting agent; in these cases it becomes the choice between two evils, and we naturally choose the least. Mr. Hutchinson, however, does not look on mercury in these cases as an evil at all, but says that by small doses the formation of red corpuscles is increased.

Another point Professor Landolt indicates is that patients are liable to travel from clinic to clinic, and, not carrying a statement of treatment with them, are very liable to be dosed over and over again with mercury.

Altogether, Professor Landolt's paper has given us much food for thought, and if it retards the reckless administration of mercury in cases not requiring it, and not likely to be influenced for good by it, it will have done an excellent work. We will conclude by quoting his own words :—

" As for myself, convinced of the absolute inutility of this treatment in the diseases cited, I consider it an imperative duty on the part of the physician to protect those who intrust to him their health against an agent which, an invaluable remedy in some cases, becomes in others a veritable poison."

And we would recommend all interested in ophthalmic practice to carefully peruse it.

